# d‐amino acids: new functional insights

**DOI:** 10.1111/febs.70083

**Published:** 2025-03-27

**Authors:** Loredano Pollegioni, Natasa Kustrimovic, Luciano Piubelli, Elena Rosini, Valentina Rabattoni, Silvia Sacchi

**Affiliations:** ^1^ The Protein Factory 2.0 Laboratory, Department of Biotechnology and Life Sciences University of Insubria Varese Italy

**Keywords:** biomarkers, d‐aspartate, d‐cysteine, d‐enantiomers, d‐serine, signaling

## Abstract

The d‐enantiomers of amino acids (d‐AAs) were initially considered “unnatural” molecules. They are primarily of microbial origin, present in low amounts, and without biological functions in eukaryotes. However, over the past few decades, sensitive analytical methods have uncovered the presence of both free and peptide‐bound d‐AAs in higher organisms. During the same period, the discovery of serine racemase—the enzyme that catalyzes the reversible formation of d‐serine from l‐serine—in rat brains demonstrated that mammals synthesize d‐AAs. Notably, the enzymes responsible for d‐AAs catabolism were identified almost 90 years ago. Subsequently, free d‐AAs such as d‐serine, d‐aspartate, d‐alanine, and d‐cysteine have emerged as a novel and important class of signaling molecules in various organs, including the brain and endocrine system. Their involvement in a wide range of neurological disorders has drawn significant scientific interest. We have focused on novel findings, based on the latest analytical techniques, that have reshaped our understanding of physiological processes across diverse organisms, from plants to humans. Beyond neurotransmission, recent studies have highlighted the versatile roles of d‐AAs in cancer, inflammation, immune regulation, kidney disease, and diabetes. Moreover, these studies suggest that the levels of d‐AAs in blood and urine could serve as early biomarkers for conditions such as Alzheimer's disease, schizophrenia, and chronic kidney disease. Understanding the role of d‐AAs in certain pathological states is helping to identify new therapeutic targets, offering promising opportunities for clinical applications in treating various diseases.

## Graphical abstract

This review explores recent advances in understanding d‐amino acids (d‐AAs) and their pivotal roles across organisms, from plants to humans. d‐AAs have been implicated in key physiological processes, including cancer, inflammation, immune regulation, kidney disease, diabetes, and nervous system function. Moreover, emerging evidence highlights their potential as early biomarkers for several pathologies. Unraveling their roles in pathology reveals novel therapeutic targets, paving the way for innovative clinical applications.

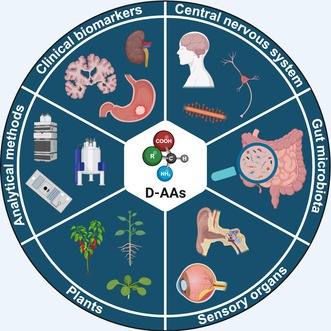

**Abbreviations**
AA: amino acid
AD: Alzheimer's disease
ALS: amyotrophic lateral sclerosis
AMPAR: α‐amino‐3‐hydroxy‐5‐methyl‐4‐isoxazolepropionic acid receptor
AMPs: antimicrobial peptides
Asc‐1: alanine‐serine‐cysteine transporter 1
ASD: autism spectrum disorder
Aβ: amyloid‐beta
BBB: blood–brain barrier
CCI: controlled cortical impact
CINs: cortical interneurons
CKD: chronic kidney disease
CRC: colorectal cancer
CSF: cerebrospinal fluid
DAAO: d‐amino acid oxidase
d‐AAs: d‐amino acids
DASPO: DDO: d‐aspartate oxidase
DSS: dextran sulphate sodium
GFR: glomerular filtration rate
HC: healthy controls
l‐AA: l‐amino acid
LOD: limit of detection
MMSE: mini‐mental state examination
MPTP: 1‐methyl‐4‐phenyl‐1,2,3,6‐tetrahydropyridine hydrochloride
N/S‐CDs@NC: N‐ and S‐doped carbon dots‐N‐rich porous carbon nanoenzyme
nhPHIP: non‐hydrogenative parahydrogen‐induced hyperpolarization
NIR: near‐infrared
NKH: nonketotic hyperglycinemia
NMDA: N‐methyl‐d‐aspartate
NMDAR: N‐methyl‐d‐aspartate receptor
Nrf2: nuclear factor erythroid 2‐related factor 2
nTRS: non‐treatment‐resistant
PD: Parkinson's disease
PFC: prefrontal cortex
PG: peptidoglycan
pkDAAO: pig kidney d‐amino acid oxidase
PP: phosphorylated pathway
PTSD: post‐traumatic stress disorder
SCZ: schizophrenia
SERS: surface‐enhanced Raman scattering
SLC: solute carrier
SNAT: sodium‐coupled neutral amino acid transporter
SR: serine racemase
Th: T helper
TRS: treatment‐resistant
UC: ulcerous colitis

## Introduction

**Fig. 1:**
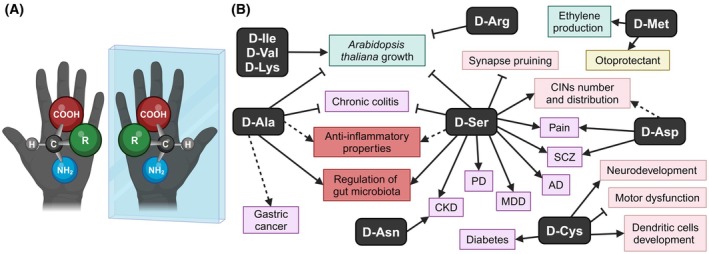
d‐amino acids: structure and recently discovered roles. (A) Representation of the l‐ and the d‐enantiomers of a general amino acid (R = side chain); (B) The most relevant discoveries on d‐AAs from 2018 reported in this review. Dashed lines indicate aspects requiring further investigation. The recent insights into d‐AAs span various fields, including plants (light blue), central nervous system physiology (pink), sensory organs (yellow), intestinal inflammation (dark orange), and diseases (purple). AD, Alzheimer's disease; CINs, cortical interneurons; CKD, chronic kidney disease; MDD, major depressive disorders; PD, Parkinson's disease; SCZ, schizophrenia.

Amino acids (AAs) are simple molecules containing both an amine and a carboxylic acid group attached to the same carbon atom (the α‐C), to which a hydrogen is always the third substituent. Hence, when the fourth substituent is not hydrogen (glycine), the α‐C is a chiral center and the α‐AA exists in two molecular configurations called l‐ and d‐enantiomers (Fig. 1A) with identical chemical and physical properties except for the way they interact with other chiral molecules and polarized light. Proteins are made by the l‐amino acids (l‐AAs): the origin of homochirality in nature (i.e. the use of a single enantiomer) is still unknown. The ubiquity of l‐AAs in proteins (and of d‐sugars in nucleic acids) across all domains of life suggests that this selection occurred before the Last Universal Common Ancestor. Primitive meteorites contain various AAs of extra‐terrestrial origin in enantiomeric excess, advocating an abiotic synthesis of AAs in a non‐racemic manner [1].

d‐Amino acids (d‐AAs) are present in some plants, fruits, and vegetables [2]. d‐AAs are also natural components in fermented foods since they can originate from the starting materials as well as from microbial activity during fermentation: d‐Asp levels reach up to 36% and 21% in beer and yogurt, respectively, and d‐Ala reaches 42% and 14% in Emmentaler cheese and carrot juice, respectively. An increasing number of foods rich in d‐AAs are produced by the food industry due to racemization during processing. d‐AAs provide a distinct taste to foods and are generally sweeter than their l‐enantiomers. No toxic effects were reported for d‐AAs when used in racemic form for parenteral nutrition of adults and children [3]. The lowest dietary intake of d‐AAs was calculated at 10–30 mg·day^−1^ (in a diet based on little processed foods and raw foods) while a typical Western diet reaches 50–100 mg·day^−1^. Furthermore, d‐AAs have been suggested as effective in promoting weight loss. For recent reviews see [4, 5].

d‐AAs are present and essential in bacteria to crosslink peptidoglycan (PG) strands, especially using d‐Ala and d‐Glu. Bacteria possess a wide variety of enzymes capable of producing and degrading d‐AAs [6]. Bacteria in the soil are also responsible for d‐AAs presence in plants [7], although plants can produce d‐AAs through endogenous enzymes [8].

Production of d‐AAs by racemases has been reported in various invertebrate species, from arthropods [9] to mollusks [10,11], from Nematoda to Annelida, Tardigrada, and Echinodermata [11, 12]. For recent reviews reporting on the state of the art in d‐AAs synthesis, see [13, 14, 15].

d‐AAs and their roles were only recently identified in mammals, mainly due to advancements in analytical techniques [16]. Following the first report of d‐Glu in cancerous tissues in 1939 [17], low to trace levels of all d‐AAs are considered ubiquitous in mammalian tissues [18]. d‐Ser is an important co‐agonist for N‐methyl‐d‐aspartate (NMDA) receptors (NMDAR), d‐Asp is present in a wide variety of mammalian tissues and cells [19, 20, 21], and d‐Glu can be converted into d‐Gln by the glial enzyme glutamine synthetase [22]; finally, d‐Cys is the newcomer in the field. In mammals, d‐AAs frequently act as neuromodulators, overturning fundamental axioms of biology [23, 24].

Many natural peptide compounds contain at least one d‐AA: 132 have been recently presented in [25], showing that 37.9% of total AAs have the d‐configuration. These compounds are mostly produced by non‐ribosomal peptide synthesis. In bacteria, d‐AAs were found in antibiotics, biosurfactants, toxins, and siderophores. Prokaryotic cyanobacteria and eukaryotic algae produce d‐AA‐containing compounds with cytotoxic activity useful as anticancer, antimalarial, or antibiotic compounds. Marine invertebrates (mollusks, sponges, and snails) also produce d‐AA‐containing compounds, as do animal cells that synthesize d‐AA‐containing neuroexcitatory and neuroprotective peptides, cardioexcitatory peptides, hyperglycemic hormones, opioid peptides, antimicrobial peptides, natriuretic and defensin‐like peptides, and fibrinopeptides [26].

Given the vast literature, this review will not attempt to address the many facets of d‐AAs in all organisms (for recent reviews see [5, 6]) but aims to provide an overview of the most recent and noteworthy discoveries from the past five years (Fig. 1B).

## d‐amino acids in plants

Over the last two decades, the d‐AAs metabolism in plants has been documented, emphasizing their role in several processes such as the interaction with soil microorganisms, signaling pathways, and regulatory mechanisms in response to the environment [27]. Several d‐AAs are found in most plants, with d‐Ala, d‐Asp, d‐Arg, and d‐Glu present in fruits (apples, grapes, and oranges) and vegetables (carrots, tomatoes, and cabbages) [28]; their endogenous levels generally represent less than 3.4% and 0.7% of total AAs content, respectively. Plants can readily absorb d‐AAs from the soil originating from chemical racemization and bacterial colonization. Different transporters for the active uptake and transport of d‐AAs have been reported, showing significant affinity and specificity for the d‐form; for a summary, see [8]. Different enzymes related to d‐AAs metabolism have been identified in various plants, thus suggesting that the plants can both produce and metabolize d‐AAs (Table 1).

**Table 1:** Enzymes involved in d‐AAs metabolism in higher plants.

Enzyme	EC number	Amino acid
d‐amino acid transaminase (from *Arabidopsis thaliana*)	2.6.1.21	d‐Met
Serine racemase	5.1.1.10	d‐ and l‐Ser
Aspartate racemase	5.1.1.10	d‐ and l‐Asp
d‐amino acid racemase (from *Arabidopsis thaliana*)	5.1.1.10	l‐Ile
d‐amino acid oxidase (from Zea maize)	1.4.3.3	d‐Ala, d‐Asp
d‐Cys desulfhydrase	4.4.1.15	d‐Cys
d‐Ala‐d‐Ala ligase	6.3.2.4	d‐Ala

From an evolutive point of view, the presence in higher plants of enzymes involved in d‐AAs metabolism is probably due to the cyanobacterial origin of plastids. Recent studies highlighted d‐AAs as structural elements: the presence of (proto) peptidoglycan in the chloroplast envelope of *Nicotiana* and *Arabidopsis* has been demonstrated [29], supporting the endosymbiont hypothesis that chloroplasts originated from cyanobacteria. Notably, treatment with d‐cycloserine, an antibiotic that affects the synthesis of the bacterial peptidoglycan by inhibiting the d‐Ala‐d‐Ala ligase, resulted in a significantly impaired *Arabidopsis* growth leading to disrupted chloroplasts [29].

d‐AAs are involved not only in nitrogen metabolism in soil, but also in plant cell growth and development [8, 30]. Studies have shown that d‐Ser, d‐Ala, and d‐Arg strongly inhibit the growth of *Arabidopsis thaliana*, while d‐Ile and d‐Val have an opposite effect, and d‐Lys promotes the growth of both *Arabidopsis* and tobacco [28]. Recently, it was reported that d‐Asp and d‐Ala strongly inhibit primary root growth in habanero pepper [31], while d‐Leu, alone or in combination with d‐Val and d‐Cys, led to an increase in the number of lateral roots.

d‐AAs also play a key role as signaling molecules in plants. In *Arabidopsis*, the role of the transaminase AtDAT1 in d‐Met stimulating ethylene production has been demonstrated [30]: d‐Met is preferentially malonylated instead of the ethylene precursor 1‐aminocyclopropan‐1‐oic acid, thus favoring ethylene production.

## d‐amino acids in the central nervous system

### The broadening of functions attributed to d‐amino acids

**Fig. 2:**
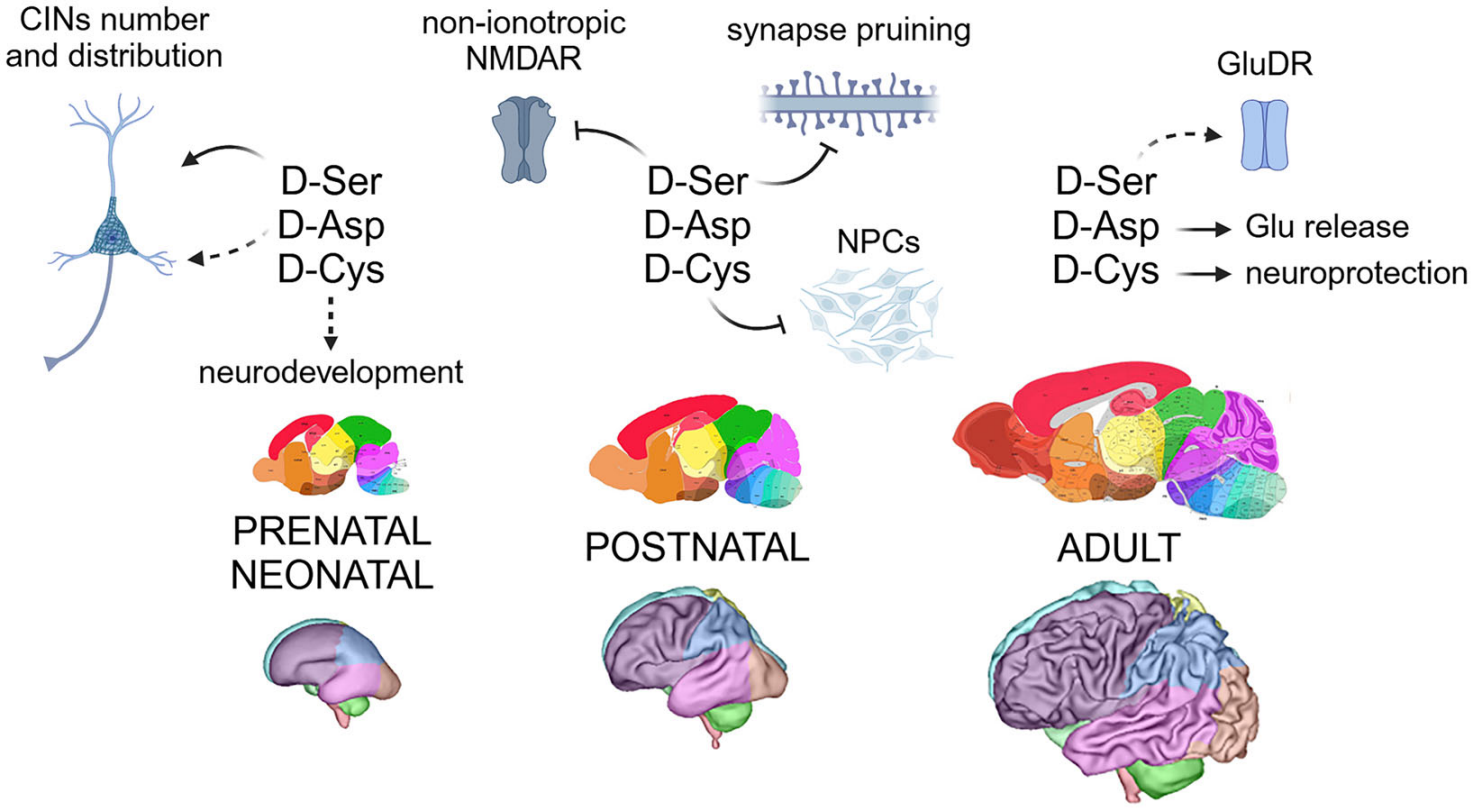
Recently established and proposed roles of d‐AAs in the brain during prenatal and postnatal development. Arrowheads indicate activation or positive regulation, while flat‐ended lines represent inhibition or negative regulation. Dashed lines denote putative novel functions that are still under investigation and require confirmation. CINs, cortical interneurons; NMDAR, N‐methyl‐d‐aspartate receptor; NPCs, neural progenitor cells; GluDR, ionotropic glutamate delta receptor.

d‐Ser binds the GluN1 and GluN3 subunits of NMDARs and acts as a channel‐opening co‐agonist [32]. It has also been proposed to modulate NMDAR downstream signaling even at rest, when the receptor is blocked by Mg^2+^: d‐Ser binding prevents intracellular conformational changes of the receptor in response to glutamate, thus blocking non‐ionotropic NMDAR‐mediated plasticity and spine shrinkage (Fig. 2) [33]. Considering that serine racemase (SR) expression in CA1 pyramidal neurons increases between the second and fourth postnatal weeks [34], elevated d‐Ser levels are likely needed to reduce the prevalence of ion flux‐independent NMDAR‐mediated plasticity following the critical period where excessive synapse pruning matches the developing nervous system to the sensory environment [33]. Studies on the SR^−/−^ mouse model underscored d‐Ser's critical role for the correct distribution of cortical interneurons (CINs): abnormal d‐Ser levels disrupt the organization of CINs leading to alterations in excitatory‐inhibitory balance at early postnatal time points, Fig. 2 [34].

Notably, d‐Ser also binds to the delta family of ionotropic glutamate receptors GluD2 and GluD1 (Fig. 2), although with far lower affinity compared to GluN1 NMDAR subunits. The two receptors are unable to bind glutamate and have been associated with the formation of synapses [35]. Very recently, a 5‐fold higher affinity for d‐Ser of GluD1 compared to GluD2 was reported [36]. In Purkinje cells and hippocampal neurons, GluD1 and GluD2 promote the formation of a tripartite complex with the presynaptic adhesion molecule neurexin via the interaction with the C1q‐like synaptic organizer cerebellins [37]. Such an arrangement regulates NMDARs and α‐amino‐3‐hydroxy‐5‐methyl‐4‐isoxazolepropionic acid receptors (AMPARs), promotes synaptogenesis, and seems essential for d‐Ser‐dependent GluD2 signaling *in vivo* [38]. A recent study, however, casts doubt on whether d‐Ser can directly gate GluD channels [39].

d‐Asp, on the other hand, acts as an endogenous agonist for NMDA and mGlu5 receptors [40, 41] and is involved in synaptic plasticity and cognitive functions, see [42]. Behavioral studies on mice indicated that the animals exposed to d‐Asp only showed a more stable memory during retrieval. The observed changes were associated with an upregulation of NMDARs and a reorganization of receptor subunit assemblies in hippocampal and dentate neurons [43]. Similarly to what was observed by microdialysis experiments in the prefrontal cortex (PFC) of freely moving mice [44], the exogenous application of d‐Asp to acute parasagittal cerebellar slices evoked the release of glutamate by Bergmann glial cells through an anionic channel‐mediated mechanism, Fig. 2 [45]. This process, in turn, triggers an excitatory signal amplification mechanism that can be activated in response to neuronal stimulation. Studies performed on a *Ddo* knockin mouse model, in which d‐aspartate oxidase (DASPO or DDO) expression starting from the zygotic stage leads to d‐Asp depletion since early developmental phases, showed no alteration in the overall composition of glutamatergic synapses. However, embryonic d‐Asp removal likely affects inhibitory cortical pathways in the medial PFC, where a significant increase in the number of parvalbumin‐positive CINs was observed, Fig. 2 [46].

d‐Cys can be rapidly produced by the spontaneous racemization of the correspondent l‐enantiomer (*t*_1/2_ = 2 days), but also by SR [47]. Its role was poorly investigated until recently, when it was proposed as a neuroprotectant in the cerebellum (Fig. 2): together with D‐amino acid oxidase (DAAO) and 3‐mercaptopyruvate sulfurtransferase, it was involved in a novel pathway that generates hydrogen sulfide and reactive sulfur species [48]. Through the generation of H_2_S, d‐Cys also affects primary cultured Purkinje cells by promoting dendritic development [49]. Moreover, it significantly ameliorates the impaired dendritic development and prevents the progression of motor dysfunction observed in an *in vitro* and *in vivo* model of spinocerebellar ataxia, respectively [50]. d‐Cys‐derived H_2_S was proposed to activate the nuclear factor erythroid 2‐related factor 2 (Nrf2) in Bergmann glia, leading to the inhibition of both astrogliosis and microgliosis in the SCA1 model mice. Moreover, the induced Nrf2 activation promoted the activity of chaperone‐mediated autophagy for protein degradation [51].

d‐Cys may also be considered an embryonic d‐AA, highly enriched in the neonatal mammalian brain (in the mm range) compared to the adult one [47]. The d‐AA level is controlled by SR and involved in neurodevelopment through reducing neural progenitor cell proliferation and homeostasis, Fig. 2. The exerted control mechanism is driven by the interaction with the myristoylated alanine‐rich C‐kinase substrate (MARCKS) and the Akt‐mediated disinhibition of the transcription factors FoxO1 and FoxO3a [47, 52, 53].

### Role of d‐amino acids in sensory organs—Physiology and rescue

d‐Ser is the principal NMDAR co‐agonist in the retina [54], with a proposed dynamic role in the recruitment of NMDARs to light intensity demands [55]. The role of the NMDARs in retinal function was recently assessed using the SR^−/−^ mice [56]: based on the animals' sex, both d‐Ser and NMDARs are involved in the field potentials of the outer retina, suggesting that gonadal hormones play a role in retinal functional integrity. Notably, abnormal levels of d‐Ser due to increased SR expression were detected in the chronic intraocular hypertension animal model, often employed to investigate glaucoma's pathophysiology [57]. Supporting its role in retina physiology, d‐Ser depletion following DAAO treatment exerted a protective effect on retinal ganglion cells in glaucomatous retina [57], involving the MEK–ERK signaling pathway [58].

d‐Ser was recently demonstrated necessary for promoting the full activation of silent NMDARs in the cochlea [59], suggesting that its signaling pathway may represent a new druggable target for treating sensorineural hearing disorders. In line with this hypothesis, the genetic depletion of d‐Ser in SR^−/−^ mice provided protection against noise‐induced long‐term damage. On the other hand, d‐Met may serve as a free radical scavenger acting as otoprotective by: (i) mitigating the oxidative stress resulting from excessive noise exposure [60]; (ii) rescuing from steady‐state and impulse noise‐induced permanent threshold shift by influencing serum antioxidant levels [61, 62]; (iii) protecting against cisplatin ototoxicity during chemotherapy [63]. In this regard, d‐Met in combination with Neurotrophin‐3 and pifithrin‐alpha was proposed to address the overwhelmed cochlear antioxidant system, the loss of neurotrophic support, and cellular activated death associated with cisplatin treatment and induced deafness [64]. Based on these findings, a d‐Met‐hyaluronan conjugated system suitable for topical delivery was developed for preventing and/or treating oxidative damage typically associated with hearing loss [65].

### New advances in d‐amino acids transporters and transport mechanisms

Beside d‐Ser, d‐Asp, and d‐Cys, also d‐Ala, d‐Arg, d‐Gln, d‐Glu, d‐allo‐Ile, d‐Leu, d‐Val, d‐Ile, d‐Lys, d‐Met, d‐Phe, and d‐Trp have been detected in trace amounts in perfused mouse brain (cortex and hippocampus), with d‐Glu unexpectedly being the least abundant [18]. The levels of these d‐AAs are 10–1000 times lower in the blood, but no specific biosynthetic pathways have been identified so far in the brain or other tissues. They could originate from gut microbiota [66] and/or from the diet, as recently demonstrated for d‐Ala [67]. Therefore, they should be absorbed by the intestine and must cross the blood–brain barrier (BBB) to reach the brain's parenchyma. At the BBB, several facilitative solute carriers have been described as AA transport systems: ASC, y^+^, X_c_^−^, LAT1, and ATB^0,+^ [68].

d‐Ser has been shown to be produced by astrocytes, neurons, or both, with compelling evidence supporting each possibility [69, 70, 71, 72]. Now it is widely accepted that, in the mammalian brain and under physiological conditions, d‐Ser is synthesized from l‐Ser in neurons [73, 74]. However, the main source of l‐Ser is from glucose via the phosphorylated pathway (PP), preferentially in astrocytes [75, 76, 77]. Recent evidence in differentiated human astrocytes indicates that the PP enzymes assemble into the “serinosome”, a metabolic multienzyme agglomerate playing a key role in controlling l‐Ser synthesis [78]. Then, glial‐derived l‐Ser is shuttled to neurons and sustains d‐Ser production, thus regulating NMDAR activity [69]. The newly synthesized d‐Ser is released by neurons via the sodium‐independent alanine–serine–cysteine antiporter 1 (Asc‐1, SLC7A10 gene product) [79, 80], belonging to the heteromeric AA transporters family. In addition, its transport through the plasma membrane is mediated by a handful of neutral AA transporters, belonging to the solute carrier (SLC) family. Both ASCT1 and ASCT2 (SLC1A4 and SLC1A5 gene products, respectively) promoted the homo‐ and hetero‐exchange of d‐Ser in rat hippocampal cultured astrocytes [81], as confirmed by the abnormally low levels of d‐Ser in the brain of ASCT1 KO mice [82].

Sodium‐coupled neutral AA transporters SNAT1 and SNAT2 (SLC38A1 and SLC38A2 gene products, respectively), and large neutral AA transporter LAT1 (SLC7A5) have also been proposed as candidate transporters. SNAT1 and SNAT2 represent a dual Gln‐d‐Ser transport system [83] mainly expressed in neurons: it controls d‐Ser concentration at synapse or peri‐synaptic sites by mediating its reuptake to prevent the full saturation and hyperstimulation of NMDARs. Notably, SLC38A5 was identified as responsible for supplying l‐Ser via the BBB for proper brain development. It is expressed in early postnatal life: the PP is insufficient for early postnatal brain development, making the supply of l‐Ser from the blood essential for d‐Ser production [84].

Concerning the mechanisms involved in d‐Ser clearance at excitatory synapses [85], ASCT1 acts as a central mediator of d‐Ser uptake by astrocytes, modulating its actual synaptic concentration. Asc‐1 also regulates the synaptic availability of d‐Ser (and Gly) [86] by undergoing changes in the conformational state and shuttling the substrate from the extracellular space to the cytoplasm [87]. The structural determinants for ligand binding and substrate specificity, as well as the molecular mechanisms that govern Asc‐1 substrate translocation by exchange and facilitated diffusion, have been recently unveiled [88, 89, 90]. For an extensive and detailed review see [5].

Two physiologically complementary systems for renal d‐Ser transport were recently identified: ASCT2 and the sodium‐coupled monocarboxylate transporters mediate the transport of d‐Ser as a noncanonical substrate [91] and contribute to d‐Ser reabsorption. On the other hand, the Asc1 transporter was reported in the derangement of serine enantiomers upon kidney injury [92]. Differently, Asc‐1 and ASCT2 mediated d‐Cys transport in HEK 293 and beta‐TC26 insulin‐secreting cell lines [52].

## d‐amino acids in the gut

Along with the diet, the microbiota has been recognized as an important source of d‐AAs [4, 93]. Gut‐derived d‐AAs are important interkingdom signaling molecules that can influence metal absorption [94], neural communication [66] and immune response [95] in the host. It is estimated that at least one‐third of the total d‐AAs in humans originate from gut microbial synthesis [96]. Although several animal studies have successfully quantified free d‐AAs concentrations within the gut lumen (summarized in Table 2), research in humans remains limited.

**Table 2:** d‐AAs identified in the gut. n/a, not applicable.

d‐AAs	Samples	Origin	Reference
d‐Ala, d‐Asp, d‐Glu, d‐Pro	Caecal samples of mice	Phylum *Firmicutes*	[103]
d‐Arg, d‐Gln, d‐*allo*‐Ile, d‐Leu, d‐Lys, d‐Met, d‐Phe, d‐Ser, d‐Trp	Gut lumen of mice	Phylum *Firmicutes*	[207]
d‐Asp, d‐Ala, d‐Glu, d‐Ser	Caecal samples of rat	Phylum *Firmicutes* (species *Bacillus*, *Staphylococcus*, *Enterococcus*, *Paenibacillus*)	[67]
d‐Ser, d‐Arg, d‐Asp, d‐Glu, d‐Ala, d‐Pro, d‐Gln, d‐Val, d‐Leu, d‐Phe, d‐Lys	Feces of WT mice	n/a	[208]
d‐Ser, d‐Pro	Feces of germ free mice	n/a	[208]
d‐Ser, d‐Asp, d‐Glu, d‐Ala, d‐Pro, d‐Gln, d‐Leu, d‐Lys, d‐Phe, d‐Val	Feces of healthy subjects and patients with UC	n/a	[97]

**Fig. 3:**
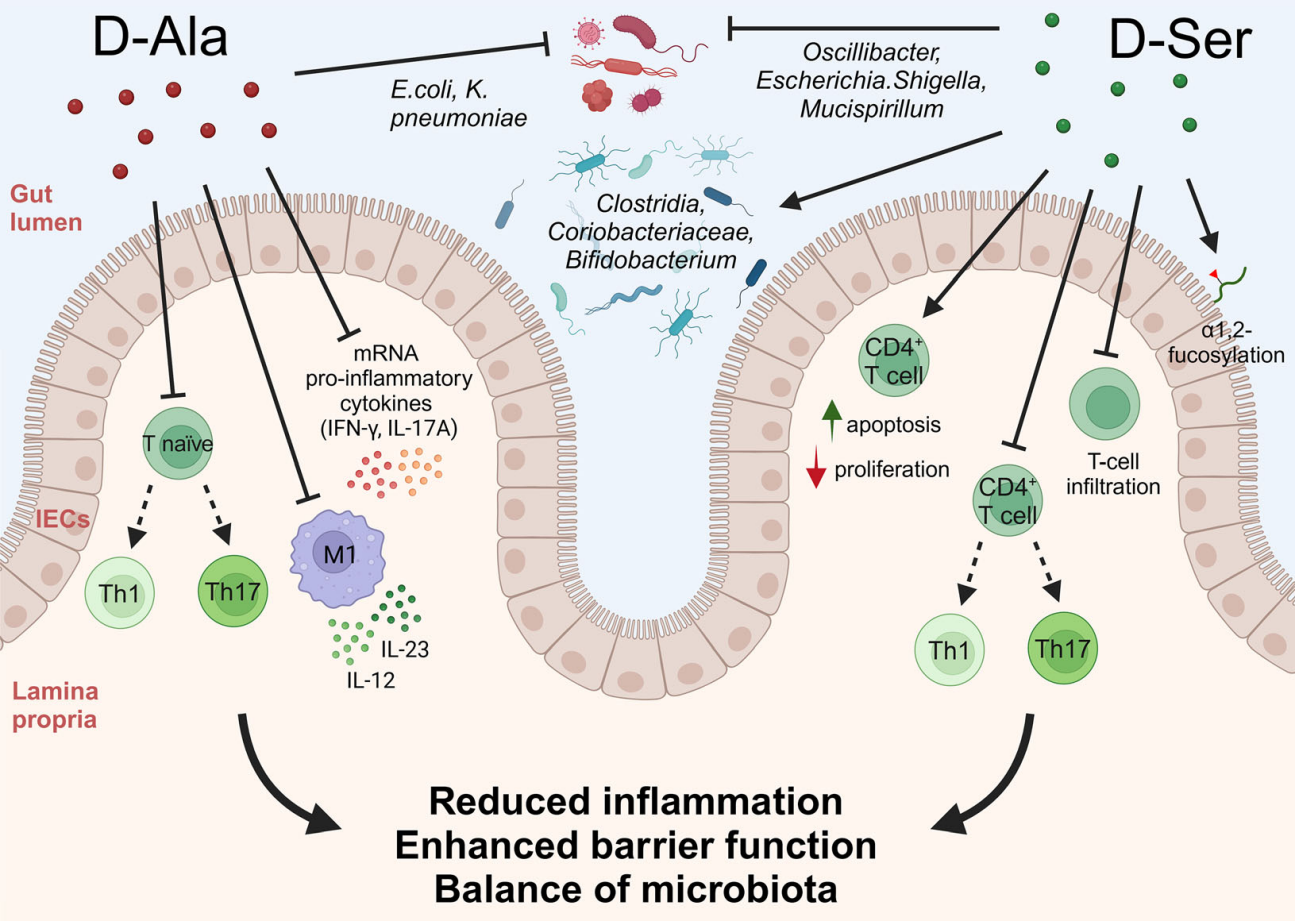
d‐AAs modulate microbial composition and influence host physiology during intestinal inflammation. d‐Ala suppresses the growth of harmful bacteria such as *E. coli* and *K. pneumoniae*, while d‐Ser reduces *Oscillibacter*, *Escherichia*/*Shigella*, and *Mucispirillum*, promoting the growth of beneficial microbes like *Clostridia UCG‐014*, *Coriobacteriaceae UCG‐002*, and *Bifidobacterium*. Both d‐Ala and d‐Ser exhibit anti‐inflammatory effects by inhibiting the differentiation of naïve T cells into pro‐inflammatory Th1 and Th17 cells. Additionally, d‐Ala reduces interleukin (IL)‐12p35 and IL‐23p19 production in bone marrow‐derived macrophages and lowers mRNA expression of key cytokines, such as interferon (IFN)‐γ and IL‐17A. d‐Ser induces apoptosis in naïve CD4+ T cells, reduces T cell infiltration, and enhances intestinal epithelial cell α1,2‐fucosylation. Collectively, these actions alleviate intestinal mucosal damage, restore dysregulated gut microbiota in experimental colitis, enhance gut barrier function, and maintain microbiota ecosystem balance.

A higher abundance of specific gut microbiota species correlates with increased levels of certain d‐AAs (summarized in Table 3), suggesting that gut bacterial composition influences d‐AAs metabolism. E.g., in patients with ulcerous colitis (UC), the fecal d‐/l‐AAs ratio, especially for Asp, Glu, Ala, Pro, and Leu, is significantly lower and coupled to an increase in the abundance of facultative anaerobes such as *Proteobacteria* [97]. In UC and intestinal inflammation, animal models and *in vitro* studies suggest that d‐AAs can modulate microbial composition and affect host physiology by actively modulating immune response (see Fig. 3). Oral administration of d‐Ser before or after colitis induction can suppress chronic colitis in Rag2^−/−^ mice by reducing T cell infiltration [98]. *In vitro*, d‐Ser inhibited activated CD4+ T cell proliferation and differentiation into pro‐inflammatory subsets of T helper (Th)1 and Th17 cells but did not affect regulatory T cell formation. d‐Ser also induced apoptosis in naïve CD4+ T cells, independent of NMDAR, Asc‐1, or DAAO pathways. Importantly, neither l‐Ser nor any of the d‐AAs tested induced the same effects observed upon d‐Ser treatment [98]. In mouse models with dextran sulphate sodium (DSS) induced colitis, d‐Ala supplementation suppressed the growth of harmful bacteria (*E. coli* and *K. pneumoniae*) alleviating colitis primarily by modulating Toll‐like receptors–MyD88 signaling [97]. Reduced serum d‐Ala levels and positive correlations of serum d‐/l‐Ala ratio with colonic inflammation were recently reported in patients with UC and in mice with DSS induced colitis [99]. Intraperitoneal injection of d‐Ala inhibited the development of colitis by decreasing the expression levels of key pro‐inflammatory cytokines in the colonic mucosa of DSS‐treated animals. d‐Ala suppressed the differentiation of naïve T cells into Th1 cells *in vitro* and reduced the production of IL‐12p35 and IL‐23p19 in bone marrow‐derived macrophages [99]. Moreover, gut microbiota‐derived d‐Ser may alleviate UC by regulating intestinal α1,2‐fucosylation [100].

**Table 3:** Correlation between d‐AAs concentrations and relative abundance of bacteria in the colon of ex‐germ free mice at the genus level. Family names of the bacteria are provided in parentheses. Most of the examined bacteria belong to phylum *Firmicutes*, class: *Clostridia*; order *Clostridiales*, apart from: *Lactococcus* and *Lactobacillus* (*Firmicutes*, *Bacilli*, *Lactobacillales*), and *Coprobacillus*, *Erysipelotrichaceae_Incertae_Sedis* and *Clostridium XVIII* (*Firmicutes*, *Erysipelotrichia*, *Erysipelotrichiales*). n/a, not applicable. Adapted from [207].

d‐AA	Negative correlation	Positive correlation
d‐Ala	n/a	*Clostridium XVIII* (*Erysipelotrichaceae*)
d‐Arg	*Erysipelotrichaceae_Incertae_Sedis* (*Erysipelotrichaceae*)	*Clostridium XVIII* (*Erysipelotrichaceae*)
d‐Asp	*Butyricicoccus* (*Ruminococcaceae*) *Marvinbryantia* (*Lachnospiraceae*) *Ananeovorax* (*Clostridiales_Incertae Sedis XIII*)	*Coprobacillus* (*Erysipelotrichaceae*) *Clostridium XVIII* (*Erysipelotrichaceae*)
d‐Gln	n/a	*Clostridium XIVa* (*Lachnospiraceae*) *Flavonifactor* (*Ruminococcaceae*) *Ananeovorax* (*Clostridiales_Incertae Sedis XIII*) *Ruminococcus2* (*Lachnospiraceae*)
d‐Glu	n/a	*Lactococcus* (*Streptococcaceae*)
d‐*allo*‐Ile	*Flavonifactor* (*Ruminococcaceae*) *Ananeovorax* (*Clostridiales_Incertae Sedis XIII*) *Clostridium XIVa* (*Lachnospiraceae*) *Oscilibacter* (*Ruminococcaceae*)	n/a
d‐Leu	*Ananeovorax* (*Clostridiales_Incertae Sedis XIII*) *Oscillibacter* (*Ruminococcaceae*)	n/a
d‐Lys	*Lactobacillus* (*Lactobacillaceae*)	*Clostridium XIVb* (*Lachnospiraceae*) *Pseudoflavonifractor* (*Ruminococcaceae*)
d‐Met	n/a	*Fusicatenibacter* (*Lachnospiraceae*)
d‐Phe	*Acetatifactor* (*Lachnospiraceae*) *Butyricicoccus* (*Ruminococcaceae*) *Oscillibacter* (*Ruminococcaceae*) *Clostridium XIVb* (*Lachnospiraceae*) *Marvinbryantia* (*Lachnospiraceae*)	n/a
d‐Ser	n/a	*Eisenbergiella* (*Lachnospiraceae*) *Clostridium XVIII* (*Erysipelotrichaceae*) *Coprobacillus* (*Erysipelotrichaceae*)
d‐Trp	*Ruminococcus2* (*Lachnospiraceae*) *Hungatella* (*Lachnospiraceae*) *Flavonifractor* (*Ruminococcaceae*)	n/a

d‐Ser and d‐Ala exhibit strong anti‐inflammatory properties, particularly in the context of intestinal inflammation [97, 99, 100]. d‐Ser reduced the abundance of bacteria positively correlating with intestinal inflammation (*Oscillibacter*, *Escherichia*, *Shigella*, *Mucispirillum*) and increased the beneficial ones [100]. Similarly, d‐Trp has also shown potential in reducing allergic airway inflammation [95]. Recent studies revealed that d‐Ser and d‐Ala can elicit pro‐inflammatory responses. In HepG2 liver cancer cells, d‐Ser reduced H_2_O_2_ production while d‐Ala had the opposite effect. Yet, both d‐AAs induced increased NF‐κB activation, TNF‐α and IL‐8 secretion [101]. Certain products of gut microbiota may exacerbate gut inflammation, compromising the intestinal barrier and increasing gut permeability [102]. This “leaky gut” may allow harmful substances to enter systemic circulation, potentially driving autoimmune disorders, metabolic dysfunctions, and chronic inflammation. Notably, while chronic HIV infection is associated with increased microbial translocation and immune activation due to the increased gut permeability, recent studies found no significant differences in plasma levels of d‐Ser, d‐Asn, d‐Pro, and d‐Ala between HIV patients and healthy controls (HC) [101].

Through DAAO, d‐AAs concur to the maintenance of gut homeostasis by regulating their own availability, shaping the composition of bacterial communities, and influencing host immune responses [103, 104]. A recent study suggested that DAAO influences gut microbiota selection through antimicrobial peptides (AMPs) generated by intestinal proteases, rather than through its catalytic activity and H_2_O_2_ production [93]. DAAO‐derived AMPs and d‐AAs were proposed to work independently in selecting gut microbiota.

## d‐amino acids and human pathologies

### d‐amino acids and diabetes

In past years, the presence of various d‐AAs in the pancreas has been established, but only d‐Ser, d‐Ala, and d‐Asp (the ones interacting with NMDARs) have been localized to islets of Langerhans and their secretions established under physiological conditions [105, 106]. For a recent review concerning the presence of free d‐AAs in endocrine glands, see [107]. d‐Ser and d‐Gln are secreted in low quantities after incubation of murine islets in 3‐ or 20‐mm glucose [108]. SR has been identified in murine and human islets and β‐cell lines [109, 110]. In glucose‐stimulated insulin secretion in mouse and human islets, d‐Cys inhibited insulin secretion more than d‐Ser; in mouse models of diabetes and non‐obese diabetes, as well as in the human pancreas, the diabetic state was characterized by high d‐Cys and SR expression levels [111]. Furthermore, lower cAMP in the pancreas, lower DNA methyltransferase enzymatic and promoter activities, followed by reduced CREB phosphorylation, were apparent in SR^−/−^ mice, yielding a decreased methylation of the *Ins1* promoter.

Diabetic retinopathy is characterized by retinal neurodegeneration and retinal vascular abnormalities, a condition affecting approx. 30% of diabetic patients with disease duration of more than 10 years. In the retina, DAAO activity is higher in the inner plexiform layer, a region rich in NMDARs, and SR is widely distributed. Actually, SR is increased in the retina of diabetic rats, and d‐Ser is increased in the aqueous humor of diabetic rats and humans [112, 113]: deficiency of SR mitigates loss of retinal ganglion cells and retinal vascular pathology in diabetic mice [114, 115].

Recently, the overexpression of DAAO before the onset of diabetes was shown to protect against neurovascular abnormalities in the retina from diabetic rats [116]. In 12‐month‐old diabetic animals, DAAO mRNA and protein levels in the retina were strongly (70%) reduced compared to controls. This correlated with increased methylation of the DAAO gene promoter and higher levels of DNA methyltransferase 1. DAAO overexpression prevented retinal ganglion cell loss and blood‐retinal barrier disruption while increasing occludin levels and reducing gliosis [116].

Of main relevance is the link between diabetes and Alzheimer's disease (AD). Hyperglycemia is a risk factor for the onset of AD, and impaired brain insulin signaling is known to occur in the disease. Low insulin response in AD has been recently linked to d‐Ser metabolism by multi‐omics analysis [117, 118], and d‐Ser may also control systemic glucose metabolism via NMDAR activation by blunting insulin secretion from pancreatic β cells [119]. Inhibition of pancreatic NMDARs, as well as loss of SR, decreased insulin secretion, a mechanistic association highlighted by single nucleotide polymorphism analysis [119]. A recent review also reported on the d‐AAs–mitochondria axis in diabetic kidney disease [120]: 30% of type 1 diabetes patients and 40% of those affected by type 2 diabetes advance to diabetic kidney disease (see below).

### d‐amino acids and cancer

In recent years, free and protein d‐AAs were investigated in both cancer and normal cells and tissues for comparison, reviewed in [121]. E.g., d‐Ala level was significantly higher in the gastric juice from gastric cancer of *H. pylori*‐positive patients [122], and d‐Glu and d‐Gln levels were lower in the serum of patients with hepatocellular carcinoma compared with HC [123]. MCF‐7 breast cancer cells showed higher levels of almost all L‐AAs compared to non‐tumorigenic MCF‐10A breast cells: d‐Asp and d‐Ser were up to 22‐fold higher in MCF‐7 breast cancer cells [124]. Notably, alteration of specific d‐AAs could represent a potential biomarker, useful for early diagnosis [125]. Very recently, d‐AAs (mainly d‐Gln) were identified in the leukemic cells from the bone narrow niche by using chiral metabolomics [126]. In saliva samples from gastric cancer patients, the d‐AAs levels were significantly higher than in healthy individuals, suggesting saliva as a suitable tool for the diagnosis of gastric cancer [127, 128, 129]. d‐AAs were also detected in urine samples from colorectal cancer (CRC) patients [130]: d‐Met level was decreased in patients with age over 50, and the d‐/l‐Gln ratio for patients at stage IV was higher than in patients at stage I. Furthermore, d‐Ser was reported to reduce the expression of the cytopathic genotoxin colibactin, which is implicated in the development of CRC [131].

Administration of selected d‐AAs inhibited tumor growth: i.e. d‐Met suppressed protein synthesis in tumor cells and also protected normal tissues, but not cultured tumor cells from radiation‐induced cell death [132, 133, 134], while d‐Cys administration inhibited cancer cell proliferation because of the generation of H_2_S by DAAO reaction [135]. Very recently, the addition of d‐AAs to the medium was reported to inhibit the proliferation of HCT116 colorectal cells [136]. d‐AAs have also been used as pro‐drugs in a DAAO‐based oxystress cancer therapy, see [137]. A further functional link between d‐AAs and cancer cell proliferation relates to the impact of NMDARs, which play significant roles during proliferation [138, 139]: certain d‐AAs reversed the antiproliferative effect caused by NMDAR channel blockers [140].

Lately, a study found a strong decrease in DAAO levels in idiopathic pulmonary fibrosis and bleomycin‐induced mouse fibrotic lung tissues [141]: d‐Ser treatment, as well as DAAO inhibition, promoted cellular senescence through the p53/p21 pathway, and the anti‐fibrotic effect of triiodothyronine depended on the DAAO levels. A recent study showed that female DDO^−/−^ mice exhibited a shorter half‐life than the wild‐type strain and developed diffuse large B‐cell lymphoma in the liver, spleen, and small intestine [142].

### d‐amino acids in neurological disorders

Neuropathic pain, a condition arising from multiple complex pathological mechanisms, is increasingly linked to d‐AAs, in both its development and persistence [24]. SR and DAAO have emerged as potential therapeutic targets for chronic pain [143] and preclinical studies demonstrated that DAAO inhibitors can reduce formalin‐induced tonic pain in mice [144].

NMDARs are central to epileptogenesis (a neurological disorder characterized by recurrent seizures), contributing to abnormal neural activity, neuronal injury, and inflammation. In animal models of temporal lobe epilepsy, d‐Ser administration prevented epileptogenesis by reducing neuron loss and gliosis, suggesting a potential neuroprotective role [145].

Controlled cortical impact (CCI) injury in mice, a model for traumatic brain injury, shifts d‐Ser release from neurons to astrocytes. Neurons downregulate d‐Ser release and astrocytes enhance its synthesis and release in the hippocampus. This shift, along with basal glutamate release, contributes to synaptic dysfunction and damage. Notably, astrocyte‐specific deletion of SR after CCI improved synaptic plasticity, brain oscillations, and learning behavior [146]. In addition, hippocampal traumatic brain injury‐induced memory deficits and dendritic spine loss are driven by prolonged d‐Ser release from both astrocytes and microglia: ablating d‐Ser synthesis or release (Slc1a4 ablation) in either cell types prevented these deficits [147].

Nonketotic hyperglycinemia (NKH) results from deficient glycine cleavage enzyme activity, leading to elevated levels of glycine in the brain. In addition to increased levels of glycine and threonine in cerebrospinal fluid (CSF, NKH patients have significantly reduced levels of serine, with a more pronounced reduction of d‐Ser [148]. Elevated glycine in NKH most likely reduces d‐Ser levels through two mechanisms: glycine inhibits SR and induces d‐Ser release via the Asc‐1 transporter [69].

### d‐amino acids in psychiatric disorders

In schizophrenia (SCZ), dysfunctions in the glutamatergic system, particularly those related to altered metabolism of d‐Ser and d‐Asp, have been well established [6, 24, 149]. A recent study revealed that approximately 30% of SCZ risk genes encode proteins located at glutamatergic synapses, influencing NMDAR function [150]. Clinically, lower serum d‐Ser levels are associated with impaired executive function in SCZ patients [151], while higher levels correlated with cognitive improvements [152]. Moreover, reduced serum d‐Asp levels were observed in both non‐treatment‐resistant (nTRS) and treatment‐resistant (TRS) SCZ patients, with a notable reduction of d‐Ser in TRS patients compared to HC [153]. d‐AA treatments have been proposed for SCZ, with d‐Ser showing a favorable safety profile in humans, even at high doses (2 g·day^−1^), though meta‐analyses indicate only modest benefits as add‐on therapy in non‐clozapine users for positive, negative, and cognitive symptoms [154, 155]. Both d‐Ser supplementation and DAAO inhibition have shown benefits in modulating mismatch negativity response, cognitive function, and extrapyramidal side effects from antipsychotic treatment [156]. Though less studied, d‐Asp may also impact SCZ, as suggested by preclinical and post‐mortem studies [157]. In a recent machine learning‐based analysis, d‐Asp/total Asp ratio and d‐Ser level in the dorsolateral prefrontal cortex distinguished SCZ patients from HC [158].

Despite the established link between glutamatergic signaling disruptions and autism spectrum disorder (ASD), no significant changes in d‐Ser and d‐Asp metabolism were reported in either cerebral tissue or serum across four ASD mouse models [159]. Another study reported comparable serum levels of d‐Ser, d‐Asp, and their l‐enantiomers between ASD patients and HC, suggesting that d‐AAs dysregulation may not be central to ASD pathology [153].

d‐Ser has been also associated with major depressive disorder. Namely, levels of d‐Ser in CSF negatively correlated with severity scores in patients [160] and mRNA expression levels of SR were altered [161]. In animal models of depression, systemic d‐Ser level enhancement—through genetic manipulation, dietary d‐Ser supplementation, or acute administration—resulted in amelioration of depressive symptoms [162].

### d‐amino acids in neurodegenerative disorders

Changes in d‐Ser metabolism appear particularly significant in multiple aspects of AD pathogenesis, reviewed in [163]. A large cohort study found a positive correlation between d‐Ser levels and AD, while d‐Asp levels remained unchanged [164]. Elevated d‐Ser levels have been observed in the brains of AD [117] and CSF of frontotemporal dementia patients [165], though other studies reported inconsistent results [166]. Increased serum d‐Ser levels in AD patients have been linked to poorer cognitive performance, suggesting peripheral d‐Ser as a useful biomarker for disease progression [164, 167]. Hyperglycemia and disrupted brain insulin signaling, known risk factors for AD onset, are associated with altered d‐Ser metabolism, as AD‐linked low insulin responsiveness affects d‐Ser regulation [117, 118]. d‐Ser may contribute to AD pathology via excitotoxic mechanisms: preclinical studies suggested that amyloid‐beta (Aβ) oligomers upregulate SR, increasing d‐Ser production and worsening neurodegeneration. Notably, SR deletion in the APP/PS1 AD mouse model reduced Aβ‐induced neuronal loss [168]. Furthermore, the rise in DAAO concentration in serum of AD patients correlated with cognitive decline severity [169]. Beyond d‐Ser and d‐Asp, levels of several other d‐AAs (d‐Ala, d‐Leu, d‐Glu, d‐Phe, d‐Pro) were also found altered in the serum of AD patients [170].

Recent research implicates d‐AAs in Parkinson's disease (PD) pathogenesis. In rhesus monkeys, 1‐methyl‐4‐phenyl‐1,2,3,6‐tetrahydropyridine hydrochloride (MPTP) treatment resulted in a d‐Ser decrease in the substantia nigra and a robust increase of d‐Asp and l‐Asp levels in the putamen, coupled to an increase of d‐ and l‐Ser levels. Interestingly, l‐DOPA treatments restored these d‐AA levels [171]. These findings were confirmed by a recent study in which both d‐ and l‐Ser were found to be elevated in the rostral putamen of MPTP‐treated monkeys [172]. PD patients not treated with l‐DOPA had lower d‐Ser levels in CSF, and l‐DOPA therapy normalized d‐Ser content [171]. Elevated d‐Ser levels were detected in the post‐mortem striatum of human PD brains, and in the CSF of newly diagnosed, untreated PD patients, when compared to individuals with other neurodegenerative diseases and HC [173]. Conversely, a recent study found no differences in the serum levels of d‐ and l‐Ser between PD and HC in age‐ and sex‐adjusted analyses. Anyway, d‐Ser and D−/total serine ratio positively correlated with age in PD, and with the age at disease onset [174].

### d‐amino acid in kidney disease

In the last 10 years, the role of d‐AAs in severe kidney diseases such as chronic kidney disease (CKD) has been substantiated [175]. Increased levels of d‐Ser and a halved d‐Ser/l‐Ser ratio were found in the serum and urine, respectively, of a mouse model of acute kidney injury already in the early phase of the disease [176]. The levels of d‐Ser, d‐Pro, and d‐Asp were strictly associated with kidney function (based on estimated glomerular filtration rate, GFR): d‐Ser and d‐Asn levels in plasma significantly increased with the progression of the disease, and the risk of developing end‐stage kidney disease was 3‐to‐4 fold higher in patients presenting higher levels of plasma d‐Ser and d‐Asn [177]. The correlation of d‐Ser concentration with the actual GFR is comparable to those of conventional kidney markers, such as creatinine and cystatin C [178], but d‐Ser level is less affected by clinical factors such as age, sex, and body weight than the conventional markers [178]. Furthermore, kidneys regulate the distribution of d‐AAs in the human body by enantioselective excretion: the d‐ and l‐enantiomer ratio was much higher in urine than in blood, and the reabsorption of d‐Ser was affected by CKD [178]. The roles of d‐AAs, and especially of d‐Ser, in renal physiology and pathology have been reviewed [175, 179].

Furthermore, a proliferative effect of d‐Ser in the proximal tubule (where d‐Ser is reabsorbed) has been observed in mice. In a mouse model of acute kidney injury, d‐Ala had a protective effect and ameliorated the symptoms [175]. Moreover, d‐AAs may have a protective effect against viral infections and have a role in immunological processes involved in kidney diseases.

## d‐amino acids as potential clinical biomarkers

Reliable biomarkers for a specific disease should show relevant alterations in affected vs. healthy subjects, be easily and accurately detectable using a cheap, robust, and time‐saving analytical method, and be observable in easily available biological samples, such as blood, saliva, or urine, not requiring invasive procedures or analyses. The clinical diagnosis of AD using biomarkers often relies on costly imaging techniques, such as PET scans, or invasive procedures like lumbar puncture. Thus, AD is still often diagnosed based on cognitive tests, causing a delay in intervention. A meta‐analysis of seven trials with about 1200 participants reported increased levels of d‐Ser in the serum of AD patients with respect to HC [167]. Later, statistically significant higher levels of d‐Ser in the serum of AD patients have been reported [164]. Moreover, both d‐Ser levels and d‐/(d + l)‐Ser ratio increased with the progression of the disease [164]. In a different study, lower plasma d‐Glu levels have been found in both AD and Mild Cognitive Impairment patients with respect to HC, and the total mini‐mental state examination (MMSE) score positively correlated with d‐Glu levels [180]. Recently, in a cohort of 37 AD patients and 34 HC, a statistically significant decrease in the serum levels of d‐Asp and d‐Pro between the two groups was observed, whereas d‐Phe levels significantly increased; a correlation between d‐Asp levels and the progression of the disease, assessed by MMSE and clinical dementia rating scales, has been found [170]. Thus, d‐Ser and d‐Asp are the most promising d‐AAs as putative biomarkers for early diagnosis of AD and its progression, but actual evidence is not conclusive.

Levels of d‐Ser in plasma of subjects affected by amyotrophic lateral sclerosis (ALS) were significantly higher than those of HC: in about half of the ALS patients of the cohort, plasma d‐Ser levels were 2 to 4‐fold higher than those of controls, although no correlations with disability, duration of disease, or age have been observed [181].

In a pilot study, basal plasma d‐Ser level positively correlated with the severity of post‐traumatic stress disorder (PTSD) symptoms, suggesting that basal d‐Ser levels in plasma could represent a putative biomarker of the severity of PTSD symptoms and a useful predictor of clinical response [125]. In addition, many studies reported a decrease of d‐Ser plasma or serum levels in SCZ patients, reviewed in [182]. However, results are still not conclusive, and further studies are necessary to assess blood d‐Ser level as a suitable marker for SCZ onset.

A putative role of d‐AAs as biomarkers has been proposed for liver [123] and gastric [129] cancer, based on levels measured in serum and saliva, respectively (see above). Finally, a fundamental role of d‐Ser in kidney pathophysiology has been highlighted recently pointing to d‐Ser blood and/or urine levels as biomarkers for severe kidney disease such as CKD, and as predictors of the prognosis of the pathology [175].

Recent works reporting d‐AAs as putative biomarkers have been reported in Table 4; see also [182, 183].

**Table 4:** Variations of amino acid enantiomer levels in different pathologies in the light of their possible use as peripheral biomarkers.

Pathology	Sample	d‐AA	Alteration	Reference
Alzheimer's disease	Plasma	d‐Glu	Decrease	[180]
	Serum	d‐Ser, d‐/(d + l)‐Ser ratio	Increase	[164]
		d‐Asp, d‐/(d + l)‐Asp ratio	Decrease (no statistical significance)	
		d‐Ser	Increase (no statistical significance)	[170]
		d‐Asp, d‐Pro	Decrease	
		d‐Phe	Increase	
Amiotrophic lateral sclerosis	Plasma	d‐Ser	Increase	[181]
Post‐traumatic stress disorder	Plasma	d‐Ser	Increase	[125]
Schizophrenia	Plasma	d‐Ser, d‐Ser/l‐Ser ratio	Decrease	[209]
	Serum	d‐Ser, Gly	Decrease	[152]
		Total (d + l)‐Ser	Decrease	[151]
		d‐/(d + l)‐Ser ratio	Increase	
Hepatocarcinoma	Serum	d‐Ile, d‐Ala, d‐Glu, d‐Gln, d‐Met, d‐Thr	Decrease	[123]
Gastric cancer	Saliva	d‐Pro, d‐Ala	Increase	[127, 128]
		d‐AAs (profile)		[129]
Chronic kidney diseases	Plasma	d‐Ser, d‐Asn	Increase	[177]
		d‐Ser		[178]
	Serum	d‐Ser	Increase	[176]
	Urine	d‐Ser/l‐Ser ratio	Decrease	

## Analytical methods

### Chromatographic methods

Chromatographic methods are among the most frequently employed analytical approaches for the enantio‐separation of AAs [184, 185, 186]. The simultaneous identification and detection of chiral AAs in human fluids, such as urine and plasma, often require the use of derivatization or labelling methods followed by separation on a reverse‐phase C18 column. The detection of trace chiral AAs in human samples has been made possible in recent years by the development of sensitive probes combined to MS/MS analysis, such as a chiral bromine‐isotope containing aldehyde probe to detect AAs in urine and plasma with a detection limit (LOD) of 67 fg and 3.9 pg for d‐Ala and d‐Ser, respectively [187]; the Marfey's derivatizing reagent (i.e. 1‐fluoro‐2,4‐dinitrophenyl‐5‐l‐alanine amide), for the simultaneous quantification of all AAs [188]; 7‐chloro‐4‐nitrobenzoxadiazole to quantify d‐AAs in the urine of AD rat models, with LOD values in the 0.1–0.5 μg·mL^−1^ range [189]; and N‐(5‐fluoro‐2,4‐dinitrophenyl)‐l‐leucinamide to quantify ten d‐AAs in brain samples in the micromolar range [190].

**Fig. 4:**
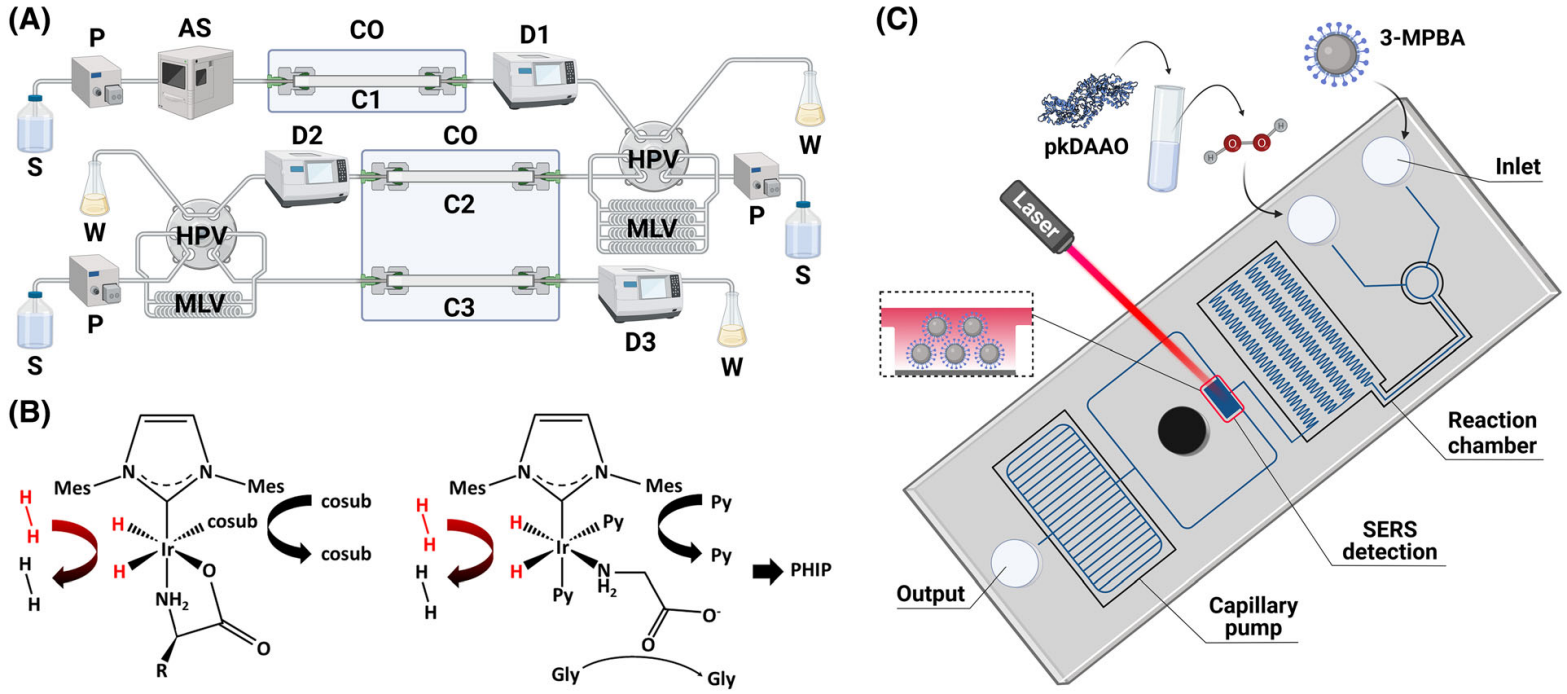
Analytical methods. (A) 3D‐HPLC system: AS, auto sampler; CO, column oven; C1, KSAARP column (1.5 mm i.d. × 500 mm); C2, KSAAAX column (1.5 mm i.d. × 150 mm); C3, KSAACSP‐001S column (1.5 mm i.d. × 250 mm); D, detector; HPV, high pressure valve; MLV, multiloop valve; P, pump; W, waste. Adapted from [193]; (B) Transient complex formed upon binding of a *α*‐AA, parahydrogen, and a co‐substrate to iridium‐heterocyclic carbene catalyst (left); schematic representation of the “amino binding” conformation geometry of glycine upon binding the catalyst in the presence of an excess of the co‐substrate pyridine to give a parahydrogen hyperpolarization (PHIP, right). Adapted from [195, 196]; (C) Surface‐enhanced Raman scattering (SERS) pump‐free microfluidic chip. Sesame Fe_3_O_4_@Au magnetic nanoparticles (SAuMNPs) with the 3‐mercaptophenylboronic acid (3‐MPBA) Raman probe were employed: 3‐MBPA is converted to 3‐hydroxythiophenol by H_2_O_2_ produced by pkDAAO, exhibiting a Raman peak at 882 cm^−1^ by oxidation of d‐AAs in the patient's saliva. The capillary pump allows an automatic flow of the fluid and the serpentine channels in the reaction zone allow for adequate mixing. Adapted from [201].

Multi‐dimensional HPLC methods enable sensitive and efficient simultaneous separation and quantification of all proteinogenic AAs across various matrices, for a review see [191]. The 2D‐HPLC system combining reversed‐phase and enantioselective columns has been applied to the determination of d‐ and l‐Phe in the human plasma and urine samples (in the 1–3 fmol to 5–15 pmol range, respectively) [192]. The addition to the system of an anion‐exchange column resulted in a 3D‐HPLC system (Fig. 4A) [193]. The derivatization with the 4‐fluoro‐7‐nitro‐2,1,3‐benzoxadiazole and the use of narrow bore columns allowed detection of trace levels of Asn, Ser, Ala, and Pro enantiomers in human plasma samples from patients affected by CDK.

Unlike liquid chromatography, ion mobility spectrometry has emerged as a promising technique for achieving isomer separation in the gas phase. In this view, an automated method has been developed by combining chiral derivatization with the (S)‐naproxen chloride probe and the trapped ion mobility–mass spectrometer separation [194]. The automated separation of different d‐AAs was achieved within 3 min per sample with a LOD value in the nanomolar range.

### NMR detection

Recently, a non‐hydrogenative parahydrogen‐induced hyperpolarization (nhPHIP) NMR method has been developed [195, 196]: it is based on the reversible binding of d‐AAs to the Ir‐IMes complex catalyst as bidentate ligands, via their amino and carboxy groups, together with parahydrogen and a suitable co‐substrate (Fig. 4B): it has been applied to human urine samples and showed a sub‐micromolar LOD [195]. A method requiring a chiral pyridine derivative as co‐substrate, such as (S)‐nicotine, allowed for the resolution of the nhPHIP‐NMR signals of d‐ and l‐AA complexes, without chromatographic fractionation [196]. This approach discriminated the enantiomeric forms of all proteinogenic AAs (except His, Met, and Cys) in human urine.

### Light‐based analytical techniques

Colorimetric, bioluminescent, and chemiluminescent methods are distinguished by their straightforward equipment and rapid analysis, as well as high sensitivity, wide linear range, and easy automation. Generally, pig kidney daao (pkDAAO) was used to oxidize d‐AAs, producing hydrogen peroxide that reacts with different compounds: (a) the N‐(4‐aminobutyl)‐N‐ethylisoluminol hydrogel system producing a strong long‐lasting chemiluminescence signal allowing detection of d‐Ala in the 1 μm‐10 mm linear range (LOD of 0.12 μm) [197]; (b) a luminescent metal organic framework terbium‐based fluorescence sensor that enabled detection of d‐AAs in human serum [198]: LOD of approx. 0.1 μm for d‐Ala and d‐Pro; (c) 3,3′,5,5′‐tetramethylbenzidine was oxidized to a blue product using carbon‐based nanoenzymes of N‐ and S‐doped carbon dots confined in N rich porous nanocarbon (N/S‐CDs@NC) [199]: d‐Pro and d‐Ala were detected with LOD of approx. 0.2 μm; (d) the CDs@NC nanozymes were successfully used through pyrolysis of ZIF‐8 precursor filled with glucose, showing a LOD of 7.7 μm and 18.6 μm for d‐Pro and d‐Ala, respectively [200]: their quantification in saliva samples was accomplished by a surface‐enhanced Raman scattering (SERS) pump‐free microfluidic chip [201], Fig. 4C; (e) the near‐infrared (NIR)‐excitable, pkDAAO‐based nanoprobe comprising four components produced an improved red luminescence signal, thus allowing a sensitive noninvasive *in vivo* d‐Ser detection (LOD of 2.2 μm) [202]; (f) a photoelectrochemical platform based on NIR conversion into visible light was constructed by combining a NaYF_4_:Yb/Er upconversion material with few‐layer TiO_2_ − Ti_3_C_2_ nanosheets and pkDAAO [203]: the biosensor detected d‐Ser in the μm range and LOD of 0.29 μm.

Noteworthy, the real‐time *in vivo* d‐Ser detection has been achieved by using a miniaturized amperometric biosensor [204] with the *Rhodotorula gracilis* DAAO [205] immobilized on a poly‐metaphenylenediamine 10 μm Pt disk microelectrode. The electrochemical biosensor showed a LOD of 0.36 μm for d‐Ser with a sensitivity of 283 ± 6 μA·cm^−2^·mm ^−1^. For details of biosensors used in d‐AAs detection, see Table 5.

**Table 5:** Comparison of analytical properties of different biosensors to detect d‐AAs in biological samples.

DAAO source	Assay technique	LOD (μm)	Detected d‐AAs	Biological sample	Reference
pkDAAO	Chemiluminescence	0.12	d‐Ala	Human serum	[197]
	Fluorescence	0.09 0.12	d‐Ala d‐Pro	Human serum	[198]
	Colorimetric	0.35 0.14	d‐Ala d‐Pro	Human saliva	[199]
	Raman spectroscopy	8.3 6.8	d‐Ala d‐Pro	Human saliva	[201]
	Luminescence	2.2	d‐Ser	Human serum	[202]
	Luminescence	0.29	d‐Ser	Human serum and rat cerebrospinal fluid	[203]
RgDAAO	Amperometric	0.36	d‐Ser	*Xenopus laevis* brain	[204]

## Conclusion

In 1874, Louis Pasteur stated “The universe is asymmetric, and I am persuaded that life, as it is known to us, is a direct result of the asymmetry of the universe or of its indirect consequences”. From our current perspective, d‐AAs play significant roles in all living organisms. The origin of d‐AAs in higher organisms can no longer be attributed solely to uptake from microorganisms. Instead, the contributions of diet and endogenous synthesis, particularly in relation to the gut microbiota, require further investigation. The newly uncovered roles of d‐AAs in human physiology are revolutionizing the field of brain science. Moreover, growing evidence of their dysregulation in major brain and metabolic diseases is paving the way for new diagnostic and therapeutic approaches across various pathologies. The physiological roles of d‐AAs in plants also warrant deeper exploration in the future.

Advancements in analytical technologies will allow for more precise assessments of d‐AA levels in biological samples, enabling an increasing number of investigations. To avoid conflicting results, we emphasize the need for appropriate controls and adherence to best practices in d‐AA determination [206]. Greater rigor and reproducibility in research methodologies will significantly accelerate progress in this field.

## Conflict of interest

The authors declare no conflict of interest.

## Author contributions

LP designed the outline of the review and critically revised the manuscript. LP, ER, SS, NK, VR, and LuPi wrote the manuscript. NK and VR prepared the figures and the tables.
